# Factors and Strategies Influencing Integrated Self‐Management Support for People With Chronic Diseases and Common Mental Disorders: A Qualitative Study of Canadian Primary Care Nurses' Experience

**DOI:** 10.1111/jan.16892

**Published:** 2025-03-10

**Authors:** Jérémie Beaudin, Maud‐Christine Chouinard, Émilie Hudon, Catherine Hudon

**Affiliations:** ^1^ Module Des Sciences infirmières, Université du Québec à Chicoutimi Québec Canada; ^2^ Faculté Des Sciences infirmières, Université de Montréal, Pavillon Marguerite‐d'Youville Québec Canada; ^3^ Faculté de Médecine et Des Sciences de la Santé, Université de Sherbrooke Québec Canada

**Keywords:** chronic diseases, common mental disorders, improvement strategies, influencing factors, integrated care, integrated self‐management support, nursing, primary care, qualitative study

## Abstract

**Aim:**

To describe the factors influencing clinical integration of self‐management support by primary care nurses for people with physical chronic diseases and common mental disorders, as well as strategies for improvement.

**Design:**

Thorne's interpretive descriptive qualitative approach.

**Methods:**

Semi‐structured interviews lasting from 60 to 90 min were carried out virtually with nurses from Family Medicine Groups and University Family Medicine Groups across the province of Quebec (Canada) from January 2022 to January 2023. Twenty‐three primary care nurses were recruited through purposive and snowball sampling from three networks. Iterative deductive and inductive thematic analysis was completed using Valentijn's Rainbow Model of Integrated Care.

**Results:**

The study identified several factors influencing integrated self‐management support from primary care nurses across integration domains: clinical (knowledge, skills, training and experience; workload; approaches and activities; attitudes and behaviours; clinical tools), professional (interprofessional and nursing roles; collaboration; team composition), normative and functional (culture and organisational mechanisms). Improvement strategies pointed to the necessity of developing training regarding common mental disorders, adapted clinical tools, clinical support and coaching through collaboration and culture change.

**Conclusion:**

These findings suggest that a cultural shift emphasising continuous improvement through targeted training and coaching is essential to enhance integrated self‐management support. Identifying factors and improvement strategies will help implement future interventions and tailor current practices.

**Implications for the Profession and/or Patient Care:**

Identifying barriers and facilitators, along with proposing improvement strategies, will enable the implementation of more effective interventions and the adaptation of care practices to better support self‐management. Additionally, it will influence stakeholders to modify the context surrounding integrated self‐management support and interprofessional practise.

**Reporting Method:**

Consolidated criteria for reporting qualitative research (COREQ).

**Patient and Public Contribution:**

No patient or public contribution.

**Summary**

- What problem did the study address?
  ○ Physical chronic diseases and common mental disorders are increasingly prevalent in primary care, necessitating a clinically integrated approach such as integrated self‐management. Therefore, identifying factors influencing this practice and strategies for improvement are crucial.
- What were the main findings?
  ○ The main findings highlighted the need for training programs focused on enhancing knowledge and skills related to common mental disorders, the development of adapted clinical tools, and the provision of clinical support and coaching. These initiatives should be fostered through collaboration and cultural change.
- Where and on whom will the research have an impact?
  ○ These findings can enhance nursing practice in primary care and influence stakeholders to modify the context surrounding integrated self‐management support and interprofessional practice.
- What does this paper contribute to the wider global clinical community?
  ○ Changes in nursing practice are needed to foster more holistic practices with populations suffering from physical chronic diseases and common mental disorders.
  ○ The development of training on common mental disorders, as well as adapted clinical tools, clinical support and coaching, through collaboration and cultural change, is recommended to improve integrated self‐management support.

## 1 Introduction

The presence of physical chronic diseases or common mental disorders is associated with an increased risk of developing one another (Scott m et al. 2007). For instance, having diabetes or cardiovascular disease exhibits a higher likelihood of experiencing depression, while those with depression are at greater risk of developing chronic physical diseases due to behavioural, physiological, and systemic factors (Daré et al. 2019). Chronic diseases are non‐communicable diseases that progress slowly over an extended period and are associated with several genetic, physiological, environmental and behavioural factors (Scott m et al. 2007). Similarly, common mental disorders, as defined by The National Institute of Health and Care Excellence (NICE), are recognised as chronic health conditions and encompass conditions such as depression, generalised anxiety disorder, social anxiety disorder, obsessive‐compulsive disorder and post‐traumatic stress disorder (National Institute for Health and Care Excellence 2011). This bidirectional association causes several negative effects on the health of individuals, such as a decrease in quality of life, an increased risk of complications and disabilities, and higher healthcare costs (Daré et al. 2019). Considering the rising prevalence of these conditions and their complex interplay, it is crucial to adopt an integrated approach to care that addresses both physical and mental health simultaneously to improve outcomes and reduce the overall healthcare burden.

Self‐management is a person's ability to actively manage their health, as well as the medical, behavioural and emotional consequences of their health problems (Lorig 2003). Self‐management requires the person to have several skills, such as problem‐solving, decision making, resource utilisation, forming a partnership with the healthcare provider, and taking action towards their health (Lorig 2003). In the province of Quebec (Canada), self‐management is one of the guiding principles for the management of chronic diseases and common mental disorders in primary care (Ministère de la Santé et des Services Sociaux 2012, 2022). As self‐management can be complex for the affected person (Gobeil‐Lavoie et al. 2019), self‐management support is an activity practiced by health professionals, such as primary care nurses, to help and empower a person to manage their health using a variety of strategies, such as health education, action planning and goal setting, and problem‐solving (Adams et al. 2004; De Longh et al. 2015). Some authors have reported the benefits of self‐management support for chronic diseases (Jonkman et al. 2016) and common mental disorders (Zimmermann et al. 2019). Current guidelines for the management of these conditions recommend the clinical integration of self‐management support for these conditions in primary care (Reynolds et al. 2018).

Self‐management support is one of the most common activities practiced by primary care nurses (Poitras et al. 2018). In the province of Quebec, primary care nurses work in family medicine groups and play an essential role with people suffering from chronic conditions. Their role covers curative, preventive, palliative and rehabilitative aspects and includes several activities, such as holistically assessing and longitudinally monitoring complex and vulnerable clienteles (Poitras et al. 2018; Swanson et al. 2020). However, despite efforts to enhance self‐management support, primary care nurses continue to face challenges integrating self‐management support for both chronic diseases and common mental disorders, focusing on either one or the other (Dineen‐Griffin et al. 2019). This issue is aggravated by organisational and systemic factors such as limited training, the fragmentation of physical and mental health care, and inadequate funding for mental health services (Dineen‐Griffin et al. 2019). It is therefore crucial to explore the factors that affect the empowerment of nurses to action integrated self‐management support and what must be addressed to ensure comprehensive care for people living with both conditions.

## 2 Background

Many guidelines (NICE management of depression in the presence of chronic diseases (National Collaborating Centre for Mental Health 2010); Diabetes Canada (Robinson et al. 2018); American Heart Association (Levine et al. 2021)) recommend clinical integration for chronic diseases and common mental disorders, which can be defined by the Valentijn et al.'s Rainbow model of integrated care (RMIC) as “coordination of person‐focused care for a complex need at stake in a single process across time, place and discipline” (Valentijn et al. 2013). Clinical integration requires a person‐focused approach, co‐creation of the care process, shared responsibility and person‐directed coordination of care (Valentijn et al. 2013). The clinical integration of self‐management support for both of these conditions offers several benefits including improved self‐management, better support for complex needs and reduced fragmentation of care (National Collaborating Centre for Mental Health 2010). However, it is still not widely applied by primary care nurses (Dineen‐Griffin et al. 2019) and clinical guidelines for primary care nursing activities remain unclear about how to clinically integrate self‐management support when these conditions are present simultaneously (National Collaborating Centre for Mental Health 2010).

Recent scoping review and qualitative study identified integrated self‐management support interventions by primary care nurses for people living with both chronic physical and mental health conditions (Beaudin et al. 2022) and reported on the experience of nurses (Beaudin et al. 2024). In the qualitative study reporting the experience of primary care nurses of integrated self‐management support (Beaudin et al. 2024), the authors defined integrated self‐management support as a holistic and humanistic person‐focused approach involving co‐creation, with an emphasis on health promotion and risk factor prevention. Integrated self‐management support was described as a personalised, person‐driven and nurse‐guided, and fosters the person's active participation. The nurse‐person relationship was critical, requiring partnership, trust‐building and respect for the person's autonomy. Continuity of care and services, responsibility sharing, as well as attitudes and behaviours conducive to integrated self‐management support were also required. It included many strategies, such as therapeutic education, biopsychosocial monitoring, goal setting, action planning and follow‐up sessions, as well as social and adherence support. One conclusion was the lack of description of the factors influencing the practice of integrated self‐management support by primary care nurses and the strategies for improvement (Beaudin et al. 2024).

Despite strong recommendations by guidelines for the clinical integration of self‐management support for both chronic diseases and common mental disorders, primary care nurses continue to face significant challenges in implementing this approach in practice. While existing guidelines emphasise the need for integrated care, they lack specific recommendations on how nurses can effectively operationalise self‐management support for individuals with coexisting physical and mental health conditions within primary care settings. Recent research has primarily focused on defining integrated self‐management support (Beaudin et al. 2022) and identifying its key components (Beaudin et al. 2024), but little is known about the factors that shape its implementation in primary care settings. Moreover, despite the recognised importance of this approach, strategies to enhance its adoption in routine primary care practice remain underexplored. A deeper understanding of these factors and improvement strategies is crucial to inform future guidelines and support the implementation of effective integrated self‐management interventions. This study aims to fill this gap by exploring the perspectives of primary care nurses on the clinical integration of self‐management support for people with coexisting chronic diseases and common mental disorders, identifying key barriers and facilitators, and proposing actionable strategies.

## 3 The Study

### 3.1 Aim

The purpose of this study was to describe the factors influencing integrated self‐management support and the strategies for improvement, asking two research questions from the nurses' perspective:

1. What are the factors influencing integrated self‐management support?
2. What are the strategies to improve the clinical integration of self‐management support for people with chronic diseases and common mental disorders?

## 4 Methods

### 4.1 Design

An interpretive descriptive qualitative study was carried out using Thorne's approach (Thorne 2016), a recognised approach for exploring a phenomenon for clinical purposes (Thorne 2016). Similar to the position of the research team, Thorne's approach is based on a constructivist and pragmatic perspective. From a constructivist standpoint, it recognises reality as constructed through the subjective experiences of individuals and incorporates the researcher's disciplinary expertise as an asset to enrich the analysis. Pragmatically, it emphasises the production of useful, practice‐oriented findings by moving beyond mere description to offer critical interpretations that address clinical care needs. Thorne also advocates methodological flexibility, drawing on different qualitative traditions while focusing research on health‐specific issues, ensuring both epistemological and methodological alignment with nursing science. Finally, a qualitative approach is also recommended when studying the factors influencing the clinical practice of an intervention such as integrated self‐management support and its improvement strategies through the experiences of nurses.

### 4.2 Study Settings and Sampling

The research project was conducted with nurses from primary care clinics, called Family Medicine Groups and University Family Medicine Groups in the province of Quebec. They consist of teams of family physicians who work in collaboration with an interdisciplinary team of nurses, social workers and other professionals to provide comprehensive services to a registered clientele, which is people of all ages with both physical and mental problems.

Two recruitment strategies were used: purposive and snowball sampling. Three networks were targeted to ensure the recruitment of nurses from various backgrounds and to have a sufficiently large pool of nurses. (1) Family medicine groups in two regions (Estrie and Saguenay‐Lac‐Saint‐Jean) of the province of Quebec (Canada) were selected because of their university affiliations and their openness to research projects. These nurses were contacted through their managers to initiate communication and obtain their contact information. (2) A nominal list of the email addresses of primary care nurses interested in participating in research projects was obtained from the Quebec Order of Nurses, the official self‐regulating body for all registered nurses and nurse practitioners in Quebec. We obtained the contact details of a targeted group of nurses after sending the ethical approval and a summary of the project to the Quebec Order of Nurses. (3) The Quebec family medicine groups nurses' virtual community of practice was also contacted through its administrator to recruit nurses. Recruitment was conducted by email for the three networks. Of the 30 nurses who expressed an interest in participating in the study, seven were excluded during the recruitment process due to a lack of experience with people with common mental disorders (*n* = 3), an inability to reach them using the contact details they provided (*n* = 3), or the participant's withdrawal during the data collection phase (*n* = 1). The recruitment period ran from January 2022 to January 2023 and ended when redundancy in the covered topics was observed (Thorne 2020). Redundancy of themes were observed around the 20th interview, and we terminated data collection after 23 interviews, as participants consistently reiterated similar perspectives and experiences without adding anything new, particularly regarding factors and strategies in the areas of clinical, occupational, normative and functional integration.

### 4.3 Inclusion and Exclusion Criteria

To be included, nurses had to: (1) speak French; (2) have at least 1 year of experience in family medicine groups; and (3) have at least one follow‐up (within the past year or still active) with individuals who had at least one concurrent chronic disease and a common mental disorder. The exclusion criteria were not to meet the inclusion criteria.

### 4.4 Theoretical Framework

**FIGURE 1:**
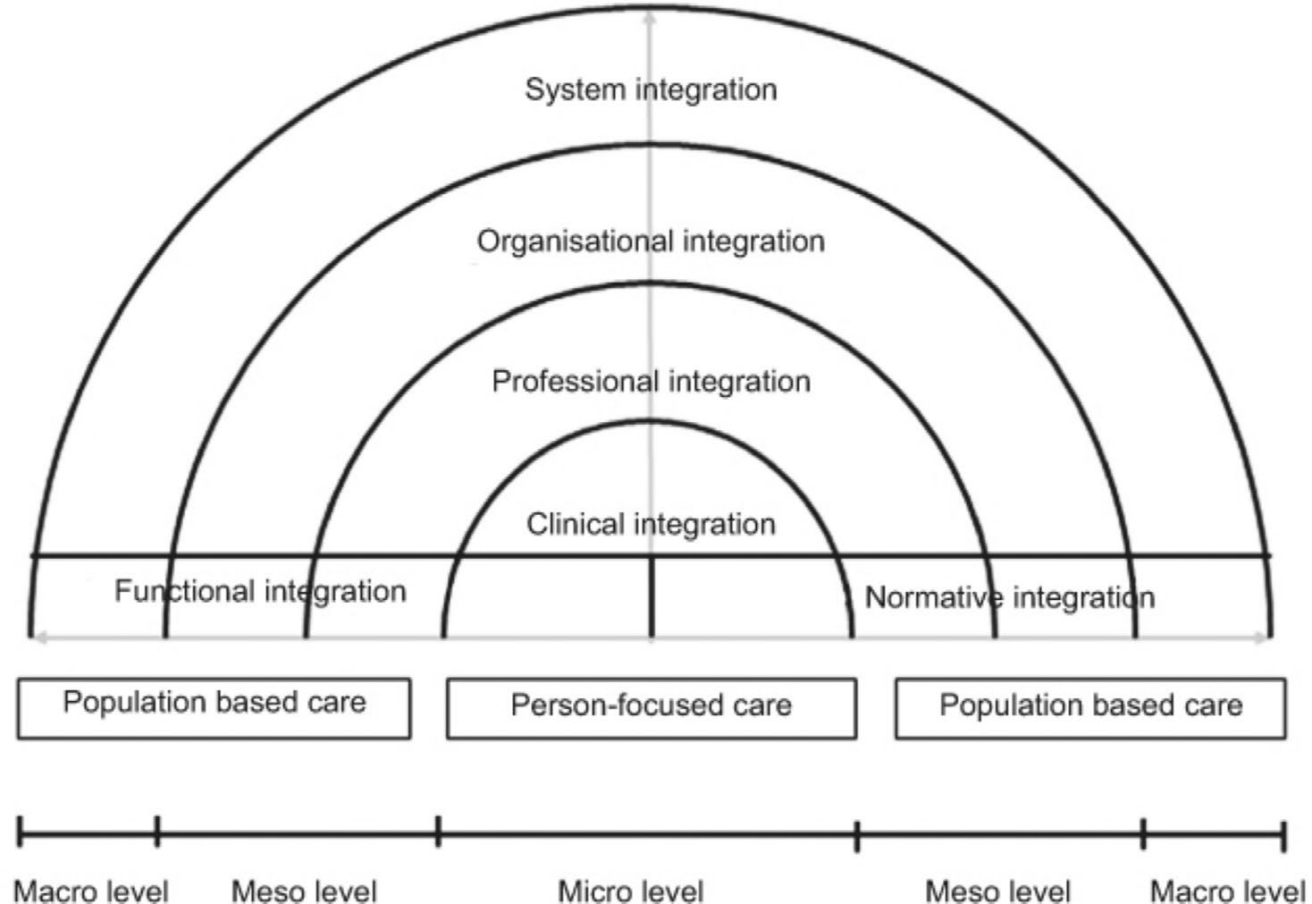
Rainbow model of integrated care. Reproduced from Valentijn et al. (Valentijn et al. 2013) in International journal of integrated care, licenced under CC BY 4.0 (https://creativecommons.org/licenses/by/4.0/). No changes were made. *Source*: https://doi.org/10.5334/ijic.886.

We used Valentijn's RMIC (Dineen‐Griffin et al. 2019) to pragmatically categorise inductively identified factors and improvement strategies under its proposed integration domains. This method is consistent with Thorne's approach, which allows the use of theoretical models as a flexible starting point during the analysis process, refined through inductive analysis (Thorne 2016). The RMIC is a conceptual model designed for primary care settings and provides a precise definition of clinical integration (Valentijn et al. 2013). The RMIC proposes several integration domains, along with the factors generally encompassed by them. The domains are designated as normative (a mutually acknowledged cultural frame of reference), systemic (policy arrangements), functional (support mechanisms and communication tools), organisational (inter‐organisational partnerships), professional (interprofessional partnerships) and clinical (see Figure 1) (Valentijn et al. 2013). In addition to clinical integration, only professional (coordination of services across various disciplines), normative (shared mission, work values, etc. within a system) and functional integration (coordination of back‐office and support functions) were retained from Valentijn's RMIC (Valentijn et al. 2013) to present results that are relevant to integrated self‐management support. Since interdisciplinary collaboration is widespread in family medicine groups, several related factors and strategies have influenced clinical integration. In addition, functional and normative integration domains are described as enablers of integrated primary care and support clinical integration (Valentijn et al. 2013).

### 4.5 Data Collection and Analysis

Taking into consideration the wide geographical distribution of Quebec, virtual, semi‐structured, one‐on‐one interviews were conducted with nurses to assess their perspective on the factors influencing their current practice of integrated self‐management support and the strategies to improve it. Although some authors note certain limitations associated with virtual interviews (e.g., confidentiality, technical difficulties) (Hensen et al. 2021), they facilitated data collection, and similar benefits and effectiveness to a face‐to‐face format have been demonstrated (Keen et al. 2022). In addition, researchers kept methodological and reflective journals throughout the study to foster reflection, and participants completed a socio‐demographic questionnaire. The Interview Guide consisted of open‐ended and follow‐up questions about barriers and facilitators to integrated self‐management support and strategies to improve its practice. The interview questions were developed in collaboration with team members using the research questions and Valentijn et al.'s (Valentijn et al. 2013) conceptual model of integrated care domains (see Data S1 file for the Interview Guide).

The guide was tested twice prior to data collection with family medicine groups nurses and modified during the study to better explore the covered topics. All interviews were conducted using Microsoft Teams (each interview lasted between 60 and 90 min (median = 70 min) and was recorded in audio and video formats) and transcribed by the first author (JB) who has a background in qualitative research and primary care nursing. To preserve a neutral and open relationship with the study's participants, the interviewer introduced himself as a student without detailing his experience. However, when questioned by a participant, the interviewer was forthright about his position, interests and reasons for carrying out the study. Data collection and analysis took place simultaneously.

Inductive and deductive thematic analysis was carried out according to Miles et al.'s (Miles et al. 2020) method using MAXQDA software. This analysis method builds on pre‐existing theories, such as the RMIC (Davies et al. 2010), while allowing for inductive in‐depth exploration of a phenomenon, which is congruent with Thorne's approach (Donnelly et al. 2020). This iterative method involved three steps: (1) data condensation, including multiple read‐throughs, writing annotations and memos, and first and second cycle coding; (2) data display using tables and modelling; and (3) drawing and verifying the conclusion. These three steps were conducted after each interview until a sufficient description of the influencing factors and improvement strategies was achieved. First, an inductive analysis (first and second cycle) using Miles et al.'s (Miles et al. 2020) method was applied during multiple read‐throughs using codes, memos and annotations. Memos and annotations were used to reflect on and clarify the interpretation of themes. For the deductive data analysis, themes and subthemes related to influencing factors and strategies for improvement that were identified from the inductive analysis were then categorised under the related integration domain from Valentijn et al.'s model (Valentijn et al. 2013). The inductive and deductive analysis allowed us to further elucidate certain previously mentioned themes of influencing factors and improvement strategies in more detail, as well as to identify and incorporate new themes. A sample coding tree is shown in the Data S1. Coding was performed by two researchers (JB and EH). Data display involved several iterations of summary tables to visualise connections and trends between codes and themes and to interpret the data. Finally, the last step (drawing and verifying the conclusion) was done through team co‐analysis (JB, EH) involving discussions about themes and the analysis process and researcher triangulation with co‐investigators (MCC, CH), which included multiple discussions about codes, themes and summary tables.

### 4.6 Ethical Considerations

The multicentre research project was approved by the CIUSSS de l'Estrie –CHUS Research Ethics Board (MP‐31‐2022‐4284; August 31, 2021). A socio‐demographic questionnaire and a consent form were sent to nurses who were interested in participating in the study through Microsoft Forms. Written consent was obtained from all participants before the interview. Participants were not offered incentives to participate, and they could withdraw at any time during the data collection process.

### 4.7 Rigour and Reflexivity

The study's design and execution were guided by Thorne's rigour criteria (epistemological integrity, representative credibility, analytic logic, interpretive authority) (Thorne 2016). Particular attention was paid to ensuring coherence between the ontological and epistemological stances of team members (pragmatism and constructivism) and the methodology used (interpretive description), thereby ensuring epistemological integrity (Thorne 2016). Representative credibility was ensured in numerous ways: Engagement with the data and throughout every step of the research project by the first author and researchers triangulation among researchers during data analysis; and the recruitment of participants from diverse backgrounds. Reflexivity through journals, ongoing reflexive memos during data analysis, and methodological transparency of the analysis process ensured analytic logic (50). The research process was meticulously documented in a methodological journal and audit trail, which facilitated the tracking of every methodological decision and ensured transparency (Miles et al. 2020). This journal enabled the first author to critically assess the research process and outcomes, considering his own subjectivity (Miles et al. 2020). Lastly, interpretive authority was ensured by clearly describing and thoroughly exploring every factor and improvement strategy; using verbatims; considering our subjectivity; and discussing all facets of the results as a team (Thorne 2016).

## 5 Results

### 5.1 Characteristics of the Sample

The final sample included 23 nurses. Table 1 presents the demographic characteristics of participants. Participants came from 11 regions across the province of Quebec with one to four nurses per region. The nurses were based in 16 family medicine groups and 7 University family medicine groups.

**TABLE 1:** Demographic characteristics of participants.

Characteristics	
Gender (*n*)	**Female: 22** Male: 1
Age (*n*)	24–34 years: 3 **35–44 years: 12** **45–54 years: 7** 55–64 years: 1
Training (*n*)	**Bachelor's degree: 22** Master's degree: 1
Years of experience (total)	**Average: 19 years** Median: 17 years (min.: 9; max.: 40)
Years of experience in family medicine groups	**Average: 9 years** Median: 9 years (min.: 1; max.: 19)
Work regime (*n*)	**Full time: 17** Part time: 6
Years working with people with chronic diseases in a family medicine group	**Average: 9 years** Median: 9 years (min.: 0; max.: 19)
Other than in family medicine groups, have you ever worked with people with chronic diseases? (*n*)	**Yes: 20** No: 3
Training received in self‐management support for chronic diseases (*n*)	**Yes: 18** No: 5
Years working with people with common mental disorders in a family medicine group	**Average: 7 years** Median: 7 years (min.: 0; max.: 19 years)
Other than in family medicine groups, have you ever worked with people with mental disorders? (*n*)	**Yes: 15** No: 8
Training received in self‐management support for common mental disorders (*n*)	Yes: 10 **No: 13**

### 5.2 Factors Influencing Integrated Self‐Management Support

The nurses' narratives helped to identify several factors influencing the practice of integrated self‐management support in the clinical domain (knowledge, skills, training and experience; workload; self‐management support approaches and activities; attitudes and behaviours; clinical tools), the professional domain (interprofessional and nursing roles; interprofessional collaboration; team composition) and the normative and functional domains (culture and organisational mechanism). Table 2 details each influencing factor inside these domains, and certain factors are detailed further in their respective sections.

**TABLE 2:** Barriers and facilitators of integrated self‐management support.

Influencing factors	Barriers	Facilitators
Clinical integration		
Knowledge, skills, training and experience	– Lack of CMD‐related knowledge and skills – Absent, obsolete or inadequate continuing education – Inadequate initial training on CMDs – Previous negative experience with people with CMDs – Insufficient training resources (time, financial, material, support) – Insufficient practice and clinical support of SMS for CMDs – Low self‐confidence in SMS abilities	– Continuing education (self‐training, higher education) and initial training on CMDs – Previous positive experience with people with CMDs – Self‐reflection on one's practice – Good interpersonal and communication skills – In‐depth knowledge of the person
Workload	– Work overload – Heavy administrative workload – Limited meeting time with the person	– Longer time for meetings and follow‐ups with the person
SMS approaches and activities	– Variable and non‐standardised practice of SMS for CMDs – Little respect for the person's autonomy – Unclear expectations – Remote follow‐up only (telephone) – Disease‐centred and directive approach	– Practice based on guidelines and conceptual models – Simple, non‐pharmacological intervention techniques – Adapted, stable, long‐term follow‐ups – In‐person or telephone follow‐up – Holistic, humane and person‐centered approach
Attitudes and behaviours	– Doubt about the person's abilities – Difficulty setting limits – Concern for the opinion of others – Fear of making a situation worse – Person's negative attitudes (e.g., closed‐minded, distrustful, restive, negative, unmotivated) or false beliefs	– Nurse's attitudes and behaviours conducive to clinical integration (e.g., versatile, motivated, self‐taught, open‐minded) – Person's positive attitudes (e.g., open‐minded, motivated)
Clinical tools	– Inadequate or absent clinical tools – Abandonment of tools (e.g., measurement scales, collective orders)	‐ Adapted clinical tools—Collective orders and protocols—Easy access to tools—Computerization of tools (e.g., computerised file, measuring scales) – Technological tools for communication and support (e.g., Teams, WhatsApp)
Professional integration		
Interprofessional roles	– Lack of role clarification – CMDs handled by other professionals	– Clear roles
Nursing role	– Low autonomy of practice – Lack of optimization of the nursing role – Broad and generalist nursing role – Nursing expertise not recognised for CMDs – Weak nursing leadership – Nursing disempowerment in the face of CMDs (e.g., not invested, referral to other professionals)	– Autonomy of practice – Pivot role – Recognition of nursing expertise for CMDs – Nursing leadership (e.g., patient advocacy, adaptability, initiative)
Interprofessional collaboration	– Low medical collaboration – Ineffective interprofessional collaboration (e.g., waiting lists, duplication and fragmentation of care, lack of follow‐up) – Difficult interprofessional communication – Work in silos	– Effective interprofessional collaboration – Interprofessional accessibility – Coaching and peer support – Regular team meeting – Effective support from clinical managers and consultants
FMG team composition	– Team instability – Lack of professionals and nurses	– Team stability – Presence of several types of professionals – Psychosocial team dedicated to FMGs
Normative and functional integration		
FMG culture	– Culture oriented towards physical health – Conservative, change‐resistant culture – Crisis (reactive) management culture	– Promotion and prevention (proactive) culture – Positive mental health culture – Collaboration culture – Teaching and continuous improvement culture – Personal empowerment culture
Organisational mechanisms	– Complex referral system – Cumbersome administration – Absent or weak support from clinical managers and consultants	– Interprofessional and inter‐organisational communication system (e.g., emails, electronic medical record) – Effective internal mechanisms (e.g., schedule management, communication, adapted access)

Abbreviations: CMD, common mental disorders; FMGs, family medicine groups; SMS, self‐management support.

#### 5.2.1 Clinical Influencing Factors

At the clinical level, a lack of knowledge, skills, training and experience with common mental disorders was noted by all family medicine group nurses:

I don't think I'm skilled enough, and I don't think I have enough knowledge. We learned all about it at university, but I never worked in mental health afterwards. I don't feel like I'm skilled enough to teach anything related specifically to [mental health], apart from general knowledge, let's say. (nurse 23)

Nurses' skills and knowledge of common mental disorders were much less developed in terms of pathophysiology, effective treatments, personality types and mental health resources. A few nurses reported having less confidence in their ability to support patients living with both chronic diseases and common mental disorders, often due to having little previous experience with common mental disorders or to having had negative experiences. However, it was clear that participating in training was often helpful. Moreover, some primary care nurses reported challenges in appropriately supporting individuals with complex needs, such as those facing cultural or linguistic barriers, low literacy or multiple chronic conditions, as well as those navigating difficult social or economic circumstances. These challenges were attributed to a lack of personal resources, such as knowledge and skills, as well as organisational and structural barriers, including insufficient training and resources and limited team support.

Training resources were deemed insufficient to meet the growing demand and needs of people with both conditions. Initial training for newly hired nurses in family medicine groups focused exclusively on physical chronic diseases. For others, ongoing training was not useful, as it remained generic and did not meet their expectations:

Our clinical coordinator provided a day's training to those who hadn't received ongoing training on mental health at about the same time as I received mine. […] my colleague went to [our clinical coordinator's training] and spent the whole day there. Then, she too left [the training] feeling a little disillusioned because she didn't feel that it met her expectations, [provide] what she needed to feel more comfortable, and then be able to start managing mental health cases. (nurse 19)

In terms of initial training and education, nurses felt that their university's bachelor's program was insufficient to prepare them to provide self‐management support for common mental disorders in primary care settings. Certain pitfalls, such as lack of time and support or accommodation from superiors to free up time, and high tuition fees, made it more difficult for nurses to complete continuing education courses. In addition, there were widespread complaints about the lack of coaching and feedback following training, or simply during clinical practice. For nurses, it was clear that receiving theoretical training was not enough to become competent in self‐management support for common mental disorders:

[The lack of mentoring] is a major gap in family medicine groups. By default, we learn a lot on our own. […] I imagine that if I spent a day with mental health nurses, I'd learn so much more than hours and hours of training. (nurse 14)

However, in terms of self‐management support approaches and activities, the nurses identified several facilitating factors. The ability to use simple non‐pharmacological interventions, such as psychological strategies focused on stress management or lifestyle management, facilitates support and enables greater autonomy of practice. Nurses stated that it helps to look beyond these people's illnesses and that taking a holistic, humane, person‐centred approach fosters a deeper connection with them:

For me, it's not so much that I'm interested in mental health, but I am interested in the person in front of me and how they feel. I can't say, ‘Wow, I love mental health’ and then start reading, no. But I see the depressed person in front of me, and it really gets to me. (nurse 12)

In addition to a more humane approach, adapting follow‐ups by offering different modalities (by telephone, in person), based on the person's needs and condition, and ensuring long‐term follow‐ups for relational continuity was also cited as a facilitator.

The majority of nurses experienced work overload, which affected the time they devoted to integrated self‐management support. Several reasons were given: shortage of nurses, busy schedules and lack of time. In this context, it was easier for nurses when more time was allocated in their schedule for these appointments. Among the approach or self‐management support activity‐related factors, nurses mentioned that self‐management support for common mental disorders is performed in a variable and intuitive way, since it is generally not guided by clear clinical guidelines, which lowers the priority of managing these conditions in favour of other types of health conditions:

When it comes to physical health, the follow‐up is determined in advance. [With diabetes collective prescriptions] when it is stable, we have glycated hemoglobin every three months, then once a year, we do a foot exam. You know, there really is a protocol, especially for diabetes, hypertension, cholesterol […]. But not for mental health, because we don't have anything official. (nurse 15)

Finally, several people pointed out that clinical tools, such as national medical protocols (such as for the management of depression or other chronic conditions), which require significant implementation work by clinical teams, were unsuitable, absent or underutilised during integrated self‐management support. Other examples included underutilisation of technological tools (e.g., phone applications; integrated measurement scales in the electronic medical records, such as Patient Health Questionnaire‐9 and General Anxiety Disorder‐7); electronic medical records that were ill‐suited to action plans; and the paucity or absence of clinical teaching tools on common mental disorders (e.g., depression, anxiety) in family medicine groups. It was clear that the availability of tools adapted to nurses' integrated self‐management support needs was a significant facilitating factor.

#### 5.2.2 Professional Influencing Factors

This domain encompassed factors related to interprofessional work within the family medicine groups, including each professional's role. Interprofessional collaboration and clarification of roles were key factors for integrated self‐management support that is consistent within the interdisciplinary team. To maintain effective collaboration between the nurse, other professionals and physicians, regular team meetings, fluid communication, easy accessibility and relational continuity between professionals were all facilitating factors. Tandem follow‐ups with a nurse and a social worker or physician were particularly helpful for integrated self‐management support. Interprofessional collaboration provided opportunities to receive coaching and peer support to optimise self‐management support. However, there were several barriers to interprofessional collaboration. Firstly, the accessibility of physicians was problematic in some cases, especially when they were on call for the hospital or working outside the primary care clinic. Another significant factor was the physicians' interest in caring for people with mental health problems. Some were more inclined to follow people with common mental disorders themselves, which could reduce the number of potential follow‐ups for nurses.

In terms of the nursing role, most nurses had a high degree of professional autonomy regarding choice of clientele, schedule management and the ability to exercise their full scope of practice. However, the broad, generalist role of family medicine groups required nurses required versatility in many areas, which reduced the expertise and time available for common mental disorders management. In addition, in the presence of other professionals authorised to follow these conditions, nurses disengaged from self‐management support of common mental disorders, directing their focus primarily towards physical chronic diseases:

As a nurse, I can give the broad outlines, but I won't provide mega‐teaching on anxiety management. If I see that there really is a problem, I have other colleagues that I can turn to. (nurse 1)

#### 5.2.3 Normative and Functional Influencing Factors

These domains encompassed everything related to family medicine groups, culture and operating mechanisms. The most important factor was the culture within family medicine groups, which played a major role in the development of nursing practice and the services offered to people with common mental disorders. A culture that promotes autonomous practice and integrated care gives nurses latitude for self‐management support, while a culture of care that focuses on organisational needs and diseases rather than on the individual makes integrated self‐management support more difficult:

Of course, for example in my family medicine group's culture, they [managers] don't really want our role to be managing that side [mental health]. [The managers] want us to talk about chronic diseases. So, if I spend the appointment talking about things related to mental health, they'll [ask questions]. That's why I would refer [the person] to the social worker or to their doctor, depending on the problem. (nurse 23)

### 5.3 Strategies to Improve Clinical Integration of Self‐Management Support

In terms of strategies to improve the clinical integration of self‐management support, several ways were proposed to respond directly to the problems experienced and cited in the previous sections. The proposed improvement strategies focus on enhancing clinical integration through targeted education, adapted clinical tools and time allocation for integrated self‐management support. Professional integration strategies emphasise role clarification, interprofessional collaboration and empowering nurses with greater autonomy and involvement in complex cases. Normative and functional integration strategies aim to foster a culture of self‐management and mental health positivity while improving organisational mechanisms, such as care pathways and team stability, to better support both conditions simultaneously. Table 3 summarises all the proposed improvement strategies, some of which are described in greater detail in the following sections.

**TABLE 3:** Improvement strategies related to influencing factors.

Influencing factors	Improvement strategies
Clinical integration	
Knowledge, skills, training and experience	– Develop nursing expertise through initial and continuing education – Reflect on practice – Develop relevant clinical experience
Workload	– Allocate more time and meetings to integrated SMS
SMS approaches and activities	– Prioritise non‐pharmacological interventions – Make more room for clinical judgement – Clearly notify the person of integrated SMS – Implement specific action plans for CMDs – Involve the family in SMS – Opt for face‐to‐face meetings for the first meeting – Develop adapted intervention strategies – Ensure interprofessional collaboration and support for people with complex needs
Attitudes and behaviours	– Adopt attitudes and behaviours conducive to integrated SMS
Clinical tools	– Develop adapted and useful tools – Free up time to develop tools and practice
Professional integration	
Interprofessional roles	– Clarify the role of each professional – Recognise expertise and work on strengths – Give more responsibility to professionals, including nurses, for CMDs
Nursing role	– Involve nurses more in complex cases – Give nurses more autonomy – Promote nurses' role and skills – Ensure nurses' versatility in physical and mental health
Interprofessional collaboration	– Develop interprofessional support and coaching – Refer more to nurses for integrated SMS – Ensure and improve medical collaboration – Ensure and improve interprofessional collaboration – Develop more interdisciplinary intervention plans – Improve interprofessional communication
FMG team composition	– Ensure stability of FMG teams (clinical and administrative) – Increase nurse‐doctor ratios in FMGs – Develop access to more professional disciplines in FMGs
Normative and functional integration	
FMG culture	– Approach mental health in a more positive way – Shift towards a culture of self‐management
Organisational mechanisms	– Establish service corridors for chronic diseases and CMDs – Clarify care and service pathways – Implement a priority‐based client triage system – Offer advanced access to the clientele

Abbreviations: CMDs, common mental disorders; FMGs, family medicine groups; SMS, self‐management support.

#### 5.3.1 Clinical Improvement Strategies

Developing nursing expertise in integrated self‐management support was the strategy most frequently raised by nurses. One proposal was the creation of integrated self‐management support orientation training for all new family medicine groups nurses, focusing on various topics specifically related to common mental disorders (e.g., pathophysiology, signs and symptoms, complications, links and impact of physical and mental comorbidity, effective treatments and support strategies, the difference between self‐management support and psychotherapy, available resources, support skills). According to the nurses, training must be adapted to the reality of family medicine groups, both in terms of format (short, accessible) and content (focused on nursing practice and the clientele). Second, some nurses suggested expanding university education to better prepare nurses for the context of primary care, given the uniqueness and rapid emergence of this practice. Beyond training, the missing link is applying the knowledge acquired during training to their practice. Another proposal was to implement mechanisms to practice integrated self‐management support, either in the form of feedback from physicians or other mental health professionals, simulation, auditing, mentoring or short‐term internships in other sectors, and especially exposure to more people living with both chronic diseases and common mental disorders:

Earlier, I was talking about suicide risk training. Well, it was really helpful, but after that, in practice, if I'm not in an environment where it happens often, if it happens once or twice a year. Well, it's like anything that you don't practice, you don't become [good]. […] At some point, theoretical training isn't enough. You must practice it, or else you put yourself in a situation. Otherwise, we forget. (nurse 17)

To develop expertise, it is necessary to allocate the required resources (budget, human resources, time). Nurses also mentioned that it was important to devote enough time to support the person's well‐being, even if this means exceeding the time allotted per meeting. Several recommendations were made regarding self‐management support approaches and activities. First, nurses recommended using clinical judgement more deliberately in decision‐making when planning follow‐ups during integrated self‐management support, rather than sticking too closely to existing protocols:

For me, I'd say it's autonomy. Letting us use our judgment. Like I said, on protocols, on what to teach, and when to stop. […] People find it [rigid] to meet every 2 weeks, 4 weeks, 6 weeks… It's very [protocol‐based]. […] Protocols are good. They're guidelines, but too much is the same as not enough. At least, that's my perception. (nurse 6)

Finally, almost all nurses highlighted the need to develop and implement clinical tools and strategies that are adapted and useful to their context. These include clinical decision support systems that integrate common mental disorders and chronic diseases; teaching, assessment and screening tools; and technological solutions such as mobile applications and telehealth. They also emphasised the importance of clinical guidelines for self‐management support and integrated care, as well as electronic templates integrated into medical records to streamline support and follow‐up for both conditions.

#### 5.3.2 Professional Development Strategies

The notion of role was at the heart of the improvement strategies. Specifically, the nurses suggested promoting and clarifying the role of family medicine groups nurses in relation to common mental disorders and enhancing the sharing of self‐management support responsibilities, based on the expertise of other professionals, especially with the social worker. Ways to foster and ensure interprofessional and medical collaboration were proposed, such as clarifying each other's needs, developing trust among professionals, and improving communication through regular team meetings. Setting up interprofessional co‐development was also proposed to foster collaboration and could include training between colleagues with complementary expertise, facilitated through meetings, a virtual platform, or the exchange of self‐training materials. It was clear to nurses that coaching and interprofessional support needed to be developed:

All that feedback. How can I put it? The work I do, I have the impression that I do it relatively well. I can see that my patients are satisfied, but I don't hear anything from the managers or the physicians. […] I don't get any feedback, whether positive or constructive, or anything like that. So, it sure would be nice to have that [feedback and coaching], because I'm suddenly thinking that if I'm doing something that I'm not supposed to or that I'm not doing properly. Well, I don't know how to adjust. (nurse 4)

#### 5.3.3 Strategies for Normative and Functional Improvement

In these domains, the main proposal was to improve organisational mechanisms such as setting up service corridors, clarifying care and service pathways, and reviewing the culture of family medicine groups, approaching mental health in a more positive and normalising way, with a vocabulary focused on psychological well‐being in the spirit of prevention and health promotion, rather than using terminology focused on mental disorders.

## 6 Discussion

This study described the factors that hinder and facilitate integrated self‐management support to examine and work on, as well as various avenues for improving the clinical integration of self‐management support for chronic diseases and common mental disorders from primary care nurses in the province of Quebec (Canada). These numerous factors were identified across various domains: clinical (knowledge, skills, training, experience; workload; self‐management support approaches and activities; attitudes and behaviours; clinical tools), professional (roles of nurses and interprofessional teams; collaboration; team composition), and normative and functional (organisational culture and mechanisms). The suggested improvement strategies aim to strengthen clinical integration through focused education, tailored clinical tools and sufficient time allocation for self‐management support. Professional integration focuses on clarifying roles, enhancing interprofessional collaboration and granting nurses greater autonomy and involvement in managing complex cases. Normative and functional integration strategies seek to promote a culture of self‐management and positive mental health attitudes, while optimising organisational mechanisms such as care pathways and ensuring stable and well‐composed teams to better help people living with both chronic physical and mental health conditions. While this study focuses on primary care organisations in Quebec, where nurses play a pivotal role in delivering self‐management support, the findings have implications for international contexts. Similar challenges, such as systemic barriers, limited training and the integration of care for chronic diseases and mental health conditions, are observed across diverse healthcare systems internationally (Tharani et al. 2021; Vallis 2015). These findings contribute to the international literature on optimising self‐management support by highlighting practical strategies that can be adapted to different organisational and cultural settings. Moreover, the emphasis on empowering nurses and fostering interdisciplinary teamwork resonates with global priorities to enhance equity and efficiency in healthcare systems (World Health Organization the United Nations Children's Fund (UNICEF) 2018).

Several of the influencing factors and improvement strategies highlighted by nurses converged on the need to develop training, clinical tools and clinical support to better support the implementation of integrated self‐management support during the initial and continuing education of family medicine group nurses. Our study is not the first to mention these needs. In their integrative review, McInnes et al. (McInnes et al. 2022) also identified nurses' significant need for primary care mental health self‐management support training, such as on depression and anxiety management, pharmacological treatments, social support and skills targeting behaviour change. These authors suggested the implementation of training programs targeting both nurses and nursing students to develop integrated self‐management support skills. Although several training programs have been developed to improve self‐management support skills among nurses (Duprez et al. 2017) and nursing students (Donnelly et al. 2020), few programs on integrated self‐management support training exist to date. Some self‐management support training programs take a more generic approach, focused on activities applicable in different contexts. For example, the INTENSS program (Duprez et al. 2022) improved knowledge, attitudes and skills in communication, and application of the 5As approach among nurses. However, these trainings require evaluation modalities such as direct observation and feedback. Another example is Ireland's Health Service Executive National Training Curriculum (Sinclair et al. 2023), which aims to train nursing students and novice nurses in self‐management support, focusing on theoretical foundations, behaviour change, communication, a holistic approach and essential skills. These generic training programmes reflect an integrated approach that transcends disease and can serve as inspiring models for integrated self‐management support training for nurses and nursing students.

In addition to training and other influencing factors identified in this study, the challenges faced by primary care nurses in delivering integrated self‐management support must also be understood within a broader systemic context. Several barriers, such as the dominance of biomedical care models in primary care and the cultural differences with mental health services, insufficient funding for mental health services, limited support for interdisciplinary collaboration and difficulties in applying specific integrated care models, reflect structural shortcomings rather than individual failures (Menear et al. 2017). Our study highlights the need for targeted policy interventions to enhance training, resources and organisational support, fostering an environment that enables nurses to deliver holistic and equitable care.

Other recurring topics included applying new knowledge to practice, especially after training, as well as clinical support or coaching, which often involved feedback. These elements are recognised as effective and are often targeted to improve self‐management support skills and quality (Duprez et al. 2017). To support nurses in their practice, clinical coaching (also known as quality improvement coaching or quality improvement collaboratives) by a team of professionals with expertise in the field, either directly associated with an organisation (such as family medicine groups) or from an external source, could be beneficial in several ways (Wells et al. 2018). Self‐management support is a complex intervention, and it is important to improve its application both to ensure quality of care and ethical practice (e.g., avoiding the risk of harm). The literature is clear on the importance of quality improvement and has suggested some approaches as effective ways to achieve it, namely the use of clinical audit (with the Plan‐Do‐Study‐Act cycle), feedback, practice facilitation and continuing education (Irwin et al. 2015). Future integrated self‐management support interventions should implement quality improvement approaches.

However, the implementation of such training or coaching interventions is complex, since it encompasses several components and involves managing and changing behaviours (Davies et al. 2018). It is necessary to plan this process and consider the factors likely to influence its implementation, which may come from both clinical and organisational levels. As recommended (Davies et al. 2010), the process of designing and implementing training for a complex primary care intervention such as integrated self‐management support should be guided by appropriate theoretical frameworks in a clearly defined implementation plan (including logic models of the problem, determinants to be considered and targeted behaviours, means to change them, and evaluation targets), and should be carried out in partnership with the people concerned. For example, the use of theoretical implementation models derived from behaviour change theories (e.g., Theoretical Domains Framework, Behaviour Change Wheel, Intervention Mapping) would be appropriate for an intervention such as integrated self‐management support (O'Cathain et al. 2019). The results of this study will enable the identification of several important barriers to integrated self‐management support, as well as strategies to address them.

Finally, this study highlights the need for self‐management support practices to go beyond individualistic approaches and incorporate a holistic view of the person and assure culturally safe practices. Ensuring that self‐management support is responsive to the cultural, social and systemic realities of Indigenous, racially minoritized and other marginalised groups is essential to engage these populations effectively and promote health equity. Many strategies to promote cultural safety in primary care, such as focusing on the patient's own goals, the use of a holistic approach, and fostering a strong and respectful relationships, are recommended (Poitras et al. 2022). Integrated self‐management support has the potential to respond more adequately to the diverse needs of marginalised populations, but more needs to be done to properly empower nurses to deliver culturally safe care at many levels. From an ethical standpoint, it is essential that nurses assure safe and equitable care among different populations, including the vulnerable and marginalised, and this will require a review of what constitutes successful self‐management support among professionals (Redman 2013). From an organisational standpoint, the culture of family medicine groups was a determining factor in the prioritisation of mental health. In Quebec, the implementation of a mental health culture is part of the ministerial mental health plan, but its application faces challenges in primary care (Menear et al. 2017). Integration of mental health promotion and prevention into primary care practices is recommended, especially through a transformation of daily practices with holistic care (World Health Organization 2022a). Beyond good training, it is necessary to move towards a culture of participation that aligns with integrated self‐management support, and this requires concerted efforts by professionals and the organisation, a true “paradigm shift” (Davies et al. 2018). An emerging, more positive approach to mental health, such as integrated behavioural health, has great potential to change the culture of care towards holistic health (Miller et al. 2016). Gaining popularity in primary care settings, this integrated care approach aims to unify physical and mental health under one organisation through clinicians known as behavioural health providers, specialising in mental health and behaviour change. In this approach, the nurse is often ideally suited to perform this role because of their holistic relational and clinical skills and their ability to facilitate care and culture change. The development of such an approach would have the potential to establish a more holistic culture of care, prevention and promotion of mental health, and would make it more feasible for family medicine group nurses to follow World Health Organisation guidelines for integrated primary care and improved self‐management of physical and mental health (World Health Organization 2022b).

### 6.1 Strengths and Limitations of the Work

To our knowledge, this is the first study to investigate influencing factors and strategies to improve the clinical integration of self‐management support for chronic diseases and common mental disorders by family medicine group nurses, from the perspective of the nurses themselves. The research team has extensive clinical and research experience in primary care and self‐management support, and the first author (JB) is a nurse with clinical experience in primary care and mental health, with a strong interest in developing nurses' skills in self‐management support for the population under study. The results come from nurses at several sites and regions across Quebec, and a rich and in‐depth description of the influencing factors and strategies was extracted with the help of verbatims, taking all facets into account, enhancing the credibility and transferability of the results to other similar primary care contexts (Miles et al. 2020). Debriefing was carried out during the analysis. Interview questions explored a wide range of factors relevant to the adoption of such a practice. Finally, the use of Valentijn et al.'s (Valentijn et al. 2013) model of integrated care domains made it possible to adequately structure the factors influencing the implementation of integrated self‐management support and the improvement strategies through these domains. That said, an implementation model could also have contributed to further analysis.

In terms of limitations, the study only looked at the experiences of nurses, and it would be relevant to explore other experiences, including those of patients. Second, most participants were nurses with several years of experience. Appointment to a family medicine group nursing position is usually achieved through the combination of an interview and accumulated years of experience (seniority within an organisation). This process may therefore explain this type of profile, and other authors have noticed this trend (Poitras et al. 2018). However, this may still have an impact on the transferability of the results to other family medicine groups' nursing practices. Transcripts were not returned to participants for comments or correction, and participants were not involved during analysis to provide feedback on the findings. Only one data collection method was used. The use of additional methods could have provided different points of view and improved confirmability and credibility (Holloway and Wheeler 2010), but the wide geographical distribution of the settings made this difficult to achieve. Some aspects of nursing practice, often common in the mental health field, may not have been covered in our study, such as outreach. Finally, while this study identified key factors and strategies for self‐management support and clinical integration, it did not explicitly explore the dimension of cultural safety in self‐management support practices of primary care nurses. A deeper exploration of cultural safety and its impact on care delivery would provide a more comprehensive understanding of how to address the needs of a diverse population with chronic physical and mental health conditions.

### 6.2 Implications for Policy and Practice

These findings can drive changes in nursing practices within family medicine groups and similar primary care settings by influencing stakeholders to adapt the context surrounding integrated self‐management support and interprofessional practice. Although focused on integrated self‐management support for chronic diseases and common mental disorders, the results may also encourage more holistic practices for patients with only one of these conditions.

Comprehensive training programmes on integrated self‐management support for primary care nurses are essential, covering topics like the pathophysiology of common mental disorders, effective treatments, mental health resources and the distinction between self‐management support and psychotherapy. Training should be integrated into both initial education and ongoing professional development. Efforts should be made to create, develop or adapt standardised clinical tools and guidelines that support integrated self‐management support in a user‐friendly, accessible and efficient manner. Digital health platforms or applications should also be considered. Addressing workload and time constraints is also critical to enabling nurses to deliver integrated self‐management support effectively, requiring special attention from policymakers and stakeholders to deliver adequate resources and supportive environments.

Interprofessional collaboration must be strengthened through role clarification, regular team meetings and support mechanisms to ensure effective communication and coordination. Ongoing clinical support and coaching for nurses, including feedback from mental health professionals, can further enhance self‐management support practices. Finally, promoting a positive culture that values mental and physical health equally is crucial, supported by policies prioritising integrated care and holistic approaches endorsed by stakeholders and organisations.

### 6.3 Recommendations for Further Research

In line with previously mentioned implications, further research should focus on developing and evaluating initial and ongoing training programmes on integrated self‐management support for people with chronic diseases and common mental disorders. Such studies could assess the effectiveness of various training models and their impact on patient outcomes. Additionally, there is a need to explore the creation and adaptation of standardised clinical tools and guidelines specifically designed for integrated self‐management support. Future research could investigate how these tools can be made user‐friendly, accessible and applicable across diverse primary care settings. The role of digital health platforms, such as mobile applications or telehealth interventions, should also be examined to determine their potential to enhance self‐management support delivery.

Future interventions should consider influencing factors and improvement strategies to overcome implementation challenges. Considering the complexity of integrated self‐management support, particular attention should be paid to the implementation plan, including the use of theoretical models to guide its implementation, such as the Model of integrated self‐management support in primary care nursing (Beaudin et al. 2024).

Secondly, interprofessional collaboration remains an understudied aera in the context of self‐management support. Future research should delve into team‐based approaches, the impact of role clarification on collaboration, and strategies for effective coaching and support among primary care teams during integrated self‐management support. Thirdly, alongside training and coaching, further investigation is needed to understand how continuous quality improvement initiatives can monitor, evaluate and optimise self‐management support and interprofessional collaboration over time, and how to ensure its sustainability in primary care settings. Finally, involving policymakers and stakeholders in future research could help reinforce the importance of integrated self‐management support within organisations, advocate for funding, and support these initiatives. Future research should also focus on patients' experiences and their needs regarding self‐management support to deepen our understanding of yet unknown influencing factors and propose ways to make it more person‐centred and equitable.

## 7 Conclusion

This study identified the factors influencing integrated self‐management support for chronic diseases and common mental disorders among family medicine groups nurses, as well as several strategies for improvement. In sum, integrated self‐management support is a complex intervention that is highly influenced by factors at the clinical, professional, normative and functional levels. A cultural shift emphasising continuous improvement through targeted training on integrated self‐management support, clinical support and coaching, team collaboration and organisational changes is essential to enhance integrated self‐management support. These results will help improve integrated self‐management support and may serve as a basis for the design of future integrated self‐management support training and clinical coaching interventions.

## Author Contributions

Jérémie Beaudin: conceptualization, methodology, investigation, formal analysis, data curation, writing – original draft, writing – review and editing, visualisation, software, project administration. Maud‐Christine Chouinard: conceptualization, methodology, validation, writing – original draft, writing – review and editing, supervision. Émilie Hudon: formal analysis, validation, writing – original draft, writing – review and editing. Catherine Hudon: conceptualization, methodology, validation, writing – original draft, writing – review and editing, supervision.

## Ethics Statement

The multicentre research project was approved by the CIUSSS de l'Estrie‐CHUS Research Ethics Board (MP‐31‐2022‐4284). All methods were carried out in accordance with relevant guidelines and regulations.

## Consent

Informed consent was obtained from all subjects and/or their legal guardian(s).

## Conflicts of Interest

The authors declare no conflicts of interest.

## Supporting information


Data S1.

## Data Availability

The datasets used and/or analysed during the current study are available from the corresponding author upon reasonable request. The data are not publicly available due to privacy or ethical restrictions.
