# Supplementary material for: Factors and Strategies Influencing Integrated Self‐Management Support for People With Chronic Diseases and Common Mental Disorders: A Qualitative Study of Canadian Primary Care Nurses' Experience
Source: J Adv Nurs. 2025 Mar 10;81(12):8754–69. doi: 10.1111/jan.16892 (PMC12623670; doi:10.1111/jan.16892)
Supplement: Supplementary file 1 — Data S1. [file JAN-81-8754-s001.pdf]

Title

Factors and strategies influencing integrated self-management support for people with chronic diseases and common mental disorders: A qualitative study of Canadian primary care nurses' experience

Supplementary file 1

Interview guide

Open-ended questions	Follow-up questions
Questions about influencing factors:<br>• What makes it easier for you to support people with physical CD and CMD?<br>• What makes it more difficult for you to support people with physical CD and CMD?	Are there factors influencing your support at the…<br>• Personal of clinical level?<br>• Professional level?<br>• Organizational level?<br>• Normative level, regarding norms or culture?<br>• Functional level?
Questions about improvement strategies:<br>• What strategies or means could be put in place to make it easier to support people with CDs and CMDs in your follow-up?	Are there strategies or means that could be put in place at the…<br>• Personal of clinical level?<br>• Professional level?<br>• Organizational level?<br>• Normative level, regarding norms or culture?<br>• Functional level?<br>If you had things to change today, what would they be?

Title

Factors and strategies influencing integrated self-management support for people with chronic diseases and common mental disorders: A qualitative study of Canadian primary care nurses' experience

Coding tree example

<table>
<tr><td>Deductive coding</td><td colspan="2">Inductive coding</td></tr>
<tr><td>Valentijn's integration domain</td><td>Influencing factors</td><td>Themes and sub themes</td></tr>
<tr><td>Clinical integration</td><td>Knowledge, skills, training and experience</td><td>Knowledge

In depth knowledge of the person (F)
Lack of knowledge (B)
On CMDs
On CMDs medication and treatments

Skills

Good interpersonal and communication skills (F)
Reflection (F)
Personal growth
Self-reflection on the approach
Pre-meeting preparation
Lack of skills (B)
For CMDs SMS

Training

Training (F)
Continuing education
Initial training
Lack of training (B)
Training cost
Lack of time to train
Scarce training resources (initial or continuing)
Lack of practical application of learning

Experience

Previous positive experience with people with CMDs (F)
Previous negative experiences with people with CMDs (B)
</td></tr>
</table>

B: Barrier; F: Facilitator
